# A comparative analysis of stably expressed genes across diverse angiosperms exposes flexibility in underlying promoter architecture

**DOI:** 10.1093/g3journal/jkad206

**Published:** 2023-09-11

**Authors:** Eric J Y Yang, Cassandra J Maranas, Jennifer L Nemhauser

**Affiliations:** Department of Biology, University of Washington, Seattle, WA 98105-1800, USA; Department of Biology, University of Washington, Seattle, WA 98105-1800, USA; Department of Biology, University of Washington, Seattle, WA 98105-1800, USA

**Keywords:** core promoter elements, constitutive promoters, TATA-box, Y patch, coreless, Plant Genetics and Genomics

## Abstract

Promoters regulate both the amplitude and pattern of gene expression—key factors needed for optimization of many synthetic biology applications. Previous work in *Arabidopsis* found that promoters that contain a TATA-box element tend to be expressed only under specific conditions or in particular tissues, while promoters that lack any known promoter elements, thus designated as Coreless, tend to be expressed more uniformly. To test whether this trend represents a conserved promoter design rule, we identified stably expressed genes across multiple angiosperm species using publicly available RNA-seq data. Comparisons between core promoter architectures and gene expression stability revealed differences in core promoter usage in monocots and eudicots. Furthermore, when tracing the evolution of a given promoter across species, we found that core promoter type was not a strong predictor of expression pattern. Our analysis suggests that core promoter types are correlative rather than causative in promoter expression patterns and highlights the challenges in finding or building constitutive promoters that will work across diverse plant species.

## Introduction

Precise control over gene expression is essential for development and survival. One of the first regulatory steps in expression regulation is transcription initiation, which is controlled by DNA regions designated as promoters. Current understanding of eukaryotic promoters is still remarkably limited, and we have difficulty even identifying a precise promoter region given an arbitrary sequence (Donczew and Hahn 2017). A core promoter region is functionally defined as the minimal region required for transcription initiation, associated with binding of RNA polymerase II (RNAPII) and general transcription factors (GTFs). Proximal and distal cis-regulatory elements contribute to the modulation of the core promoter's activity and give it its characteristic expression profile. A sequence containing the proximal cis-regulatory elements as well as the core promoters is often referred to as the “promoter” region (Biłas *et al.* 2016; Haberle and Stark 2018; Andersson and Sandelin 2020; Schmitz *et al.* 2022). In practice, cloning and analysis projects often pick an arbitrary length (e.g. up to 2,000 base pairs or until the next coding sequence) upstream of the transcription start site (TSS) to define as the promoter region (Andersson and Sandelin 2020; Schmitz *et al.* 2022).

Many core promoter elements have been identified within the core promoter region, which are important in directing RNAPII and determining the TSS. The TATA-box motif is the most well-understood of the core promoter elements, yet TATA-box-containing promoters only account for about 20% of eukaryotic promoters and about 30% of *Arabidopsis* promoters (Molina and Grotewold 2005; Donczew and Hahn 2017). In plants, additional core promoter types were proposed by Yamamoto et al. (2007, 2009) based on their identification of overrepresented motifs around a fixed distance from the TSS. Y patch, or pyrimidine patch, motifs are C and T rich motifs whose presence had been recently shown experimentally to associate with stronger expression (Jores *et al.* 2021). CA and GA are additional core promoter elements, represented in approximately 20 and 1% of genic promoters, respectively (Yamamoto *et al.* 2009). Unlike the TATA-box, which has a known GTF-binding protein associated with it, the molecular mechanisms of the Y patch, CA, and GA elements remain largely unknown. Core promoters that do not contain any of the identified core promoter types have been termed Coreless (Yamamoto *et al.* 2009, 2011). In *Arabidopsis*, Coreless promoters tend to be expressed more weakly but more broadly than those that contain TATA-boxes (Yamamoto *et al.* 2011; Das and Bansal 2019).

Constitutive promoters, defined here as promoters that are expressing in all tissues at all times, are versatile tools in synthetic biology due to their desirable expression pattern (Yang and Nemhauser 2022; Zhou *et al.* 2023). They are often used to drive expression of components used in synthetic circuits or metabolic engineering (Wu *et al.* 2014; South *et al.* 2019; Patron 2020; Brophy *et al.* 2022). Core promoter regions of constitutive promoters (such as the Cauliflower Mosaic Virus 35S promoter) have often been used as the starting point to build synthetic promoters by introducing natural cis-elements or synthetic TF-binding sites upstream of these core promoter regions to artificially tune expression strength or confer new expression patterns (Brückner *et al.* 2015; Ali and Kim 2019; Belcher *et al.* 2020; Cai *et al.* 2020; Brophy *et al.* 2022; Moreno-Giménez *et al.* 2022). However, a lack of understanding of the design constraints around promoters had made engineering synthetic promoters challenging. Current approaches often require trial and error or high throughput screening to identify functional synthetic promoters (Brückner *et al.* 2015; Belcher *et al.* 2020; Cai *et al.* 2020; Brophy *et al.* 2022; Moreno-Giménez *et al.* 2022). A better understanding of the contributions and limitations of core promoters in controlling expression patterns can therefore be essential in engineering better synthetic promoters.

Here, by leveraging publicly available RNA-seq atlases of 15 angiosperms, we were able to map gene expression pattern onto core promoter type in multiple genomic contexts. While TATA-box-containing promoters are overrepresented in conditionally expressed genes in all of the species we examined, the pattern for Coreless promoters was less clear. In most eudicots, Coreless promoters were overrepresented in uniformly expressed genes, but the opposite trend was observed in monocots. Additionally, by identifying orthologous gene groups within these species, we were able to track changes in core promoter type and expression pattern for groups of evolutionarily related promoters. We found that stably expressed genes are also more likely to have orthologs in other species compared to unstably expressed genes, and the orthologs tend to retain similar expression patterns. Last, we show that changes in core promoter types do not explain changes in expression pattern. This evolution-guided approach reveals design rules surrounding core promoter architecture and expression patterns.

## Methods

### Phylogenetic tree

A phylogenetic tree was constructed referencing NCBI's Taxonomy Browser and Li *et al.* 2021.

### RNA-seq dataset processing

*(Relevant files: 0_Slurm_Pipeline)*

RNA-seq atlases were located in the NCBI Sequence Read Archive database. The references for the datasets can be found in Supplementary Table 1. The individual datasets were retrieved using sratoolkit-3.0.1 prefetch followed by fasterq-dump functions. Fastqc-0.11.9 were used to generate a QC report for each dataset. Trimmomatic-0.39 were used for adaptor and low-quality ends trimming using the following settings: “SLIDINGWINDOW:4:20 MINLEN:36.” ILLUMINACLIP files TruSEq3-PE-2.fa were supplied for paired end data and TruSEq3-SE.fa were supplied for single end data. Reference transcriptomes were downloaded from Ensembl Plants (http://plants.ensembl.org/index.html) for *Arabidopsis thaliana, Camelina sativa, Cucumis melo, Glycine max, Phaseolus vulgaris, Pisum sativum, Vigna unguiculata, Sorghum bicolor, Zea mays, Solanum lycopersicum, Actinidia chinensis, Triticum aestivum* and Phytozome (https://phytozome-next.jgi.doe.gov) for *Arachis hypogaea, Cicer arietinum, and S. tuberosum* (Goodstein *et al.* 2012; Cunningham *et al.* 2021). An index file was generated, and the reads aligned and counted using Kallisto-0.44.0 with “-o counts -b 500.” For single end data, fragment length and standard deviation were required, but the information is difficult to locate, and so a default value of “-l 200 -s 20” was used across the board.

Another Fastqc was performed on the trimmed files, and a final MultiQC-1.13 was run on the entire folder encompassing all the log files that Fastqc, Trimmomatic, and Kallisto generated. The MultiQC report was inspected to ensure the trimming step improved read quality and there were no major warnings.

### Normalizing count, calculating CV, and percent ranking

*(Relevant files: 1_Metadata_from_RUNselector.Rmd, 2_MOR_Normalization.Rmd)*

Using an R script, the raw counts for each species were normalized using the DESeq2 package using a metadata file curated from the original study for the RNA-seq datasets. The coefficient of variation (CV) across all samples for a given atlas was used as a metric for stability for each gene, and the percentile ranking for each gene was calculated. The geometric mean for each gene was also calculated across all samples.

### Extracting intergenic region and 5′UTR

*(Relevant files: 3_ExtractPromUTR(ALL_Transcripts).ipynb, 8_ExtractPromUTR(Orthologs).ipynb)*

Gff3 annotation files and reference genomes were downloaded from Ensembl or Phytozome depending on where the reference transcriptomes were retrieved from. Forty percent of transcripts were selected from the total transcriptome, and their intergenic region and 5′UTR were extracted from the Gff3 annotation. Intergenic region and 5′UTRs of identified orthologs were extracted in a similar manner.

### Labeling core promoter types

*(Relevant files: 4_Label_Promoters.Rmd, 9_Motif_Scan.Rmd, 10_Octamer_Scan.ipynb)*

Motif scan: Intergenic regions and 5′UTR sequences are trimmed to those regions to be scanned for each core promoter types: TATA box (−100 to TSS), Y patch (−100 to +100), and Inr (−10 to +10). Intergenic regions shorter than 100 bp were excluded from analysis. Each region was scanned for their respective motifs using motif files as well as methods outlined in Jores *et al.* (2021). A motif is considered to be present when the relative motif scores are above 0.85.

Octamer scan: Intergenic regions and 5′UTR sequences are trimmed based on the positions relative to the TSS outlined in Yamamoto *et al.* (2009) (TATA, −45 to −18; Y Patch, −50 to +50; CA, −35 to −1; GA, −35 to +75). Each region was scanned for the presence of octamer motifs from the TATA, Y patch, GA, and CA lists outlined in Yamamoto *et al.* (2009). If the specified region contained at least one motif for a given promoter type, it was labeled as positive.

### Ortholog analysis

*(Relevant files: 5_At_gene_ranking.Rmd, 6_Identifying_orthologs.Rmd, 7_Processing_orthologs.Rmd)*

The *Arabidopsis* transcriptome was filtered to only include primary transcripts, and mitochondria as well as chloroplast transcripts were removed. Top 5% stable genes by CV, bottom 5% stable genes by CV, and a random set of 1,343 genes (5%) were randomly selected.

Using biomaRt in R, the Ensembl and Phytozome databases were queried for orthologs for the selected set of *Arabdiopsis* genes for each species (Durinck *et al.* 2009). Orthologs from *A. hypogaea, C. arietinum,* and *S. tuberosum* were retrieved from Phytozome, and the rest of the species from Ensembl. For an analysis in Fig. 3b, significance tests were done by ANOVA followed by Tukey's HSD (honestly significant difference). For each target gene that matched to an *Arabidopsis* transcript, only the highest expressing transcript was kept. If an *Arabidopsis* transcript retrieved more than one orthologs from a target species, these pairs of orthologs were removed from analysis. We only kept orthologous gene groups that had a “change” in expression pattern, defined as crossing the 50th percentile CV, in 2 target species, and the remaining candidates were manually mapped onto the phylogenetic tree to identify gene groups that had changes in expression patterns that are consistent with the tree. This means having changes in expression patterns that are mostly found in the same clade. Gene trees were built for these candidates using blast-align-tree (https://github.com/steinbrennerlab/blast-align-tree), and the candidate lists were further trimmed based on the gene trees to ensure a 1:1 relationship between all members in the gene group.

## Results

**Fig. 1.**
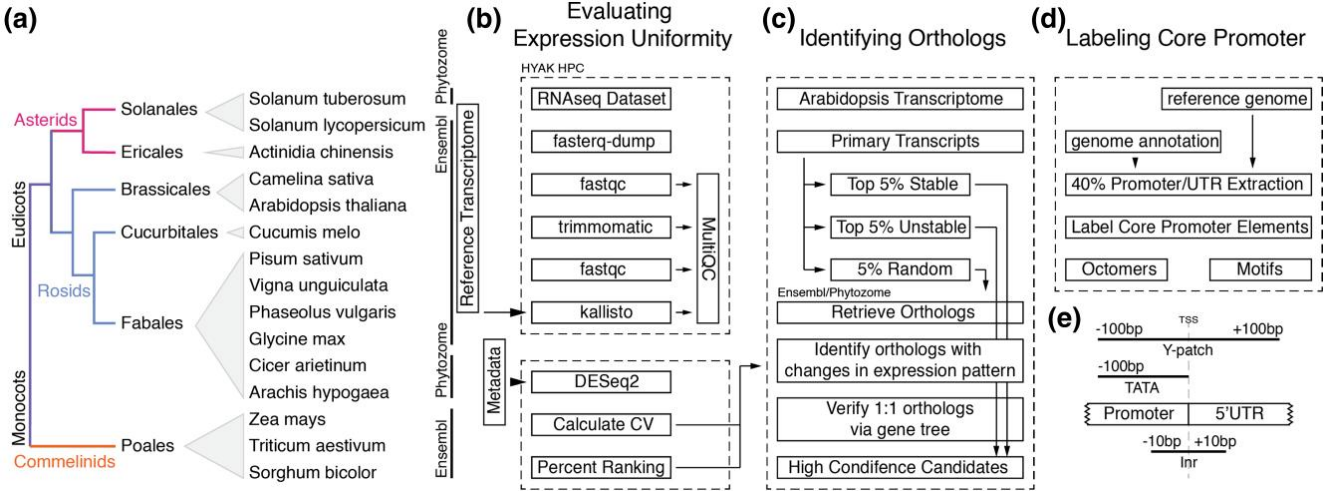
An outline of the bioinformatics pipelines. a) The 15 angiosperms included in this study and their phylogenetic relationship. b–d) The 3 major data processing steps performed in the study. Detailed parameters are included in the *Methods* section. Reference genomes, transcriptomes, and gene orthologs were retrieved via either Ensembl (Cunningham et al. 2021) or Phytozome (Goodstein et al. 2012) databases depending on the species. e) Regions searched for each core promoter motif.

We began this project by identifying species with RNA-seq Atlases, which we defined as datasets containing at least 10 different tissue samples and with samples that represented at least 2 distinct developmental stages. Although RNA-seq measures RNA levels and thus can be affected by posttranscriptional regulation, it is the best available proxy for transcriptional activity (Wang *et al.* 2009). Details regarding the dataset and their references can be found in Supplementary Table 1 (Libault *et al.* 2011; Kaeppler *et al.* 2012; Stelpflug *et al.* 2012; Potato Genome Sequencing Consortium *et al.*, 2013; Loraine *et al.* 2013a, 2013b; Moscow State University 2014; Kagale *et al.* 2014; Peanut Genome Consortium 2015; Sudheesh *et al.* 2015; Klepikova *et al.* 2016b; Vlasova *et al.* 2016; Kudapa *et al.* 2017; University of Tsukuba 2017; Yao *et al.* 2017; McCormick *et al.* 2017a, 2017b, 2017c; Penin *et al.* 2018; Ramírez-González *et al.* 2018; Brian *et al.* 2021). Figure 1a shows a phylogenetic tree of the 15 species that fit our criteria, which spans a range of angiosperms including multiple monocots and eudicots. The datasets were processed through a custom pipeline (Fig. 1b–d). In brief, Kallisto was used for RNA-seq quantification, and MultiQC was used to summarize all the outputs up till DESeq2 (Supplementary Data 1) (Bray *et al.* 2016; Ewels *et al.* 2016). For each species, normalized counts from each tissue were then converted to expression uniformity information using the CV as a metric. In this analysis, lower CV corresponds to more uniform expression, meaning comparable expression in all tissues. Higher CV, on the other hand, means less uniform and more tissue-specific expression. To facilitate comparison between species, we used percentile rank of CV as the primary metric, which represents the percentage of CVs that are less than or equal to a given value.

To determine whether the characteristic differences in expression patterns between different core promoter types seen in *Arabidopsis* hold across all the species in our dataset, we extracted the −100 bp to +100 bp region around the TSS as the “core promoter region” for 40% of all promoters in each species (Fig. 1d). TATA-box, Y patch, and Inr motifs were screened according to the methods detailed in Jores *et al.* (2021). The regions scanned for each motif are more relaxed than their known regions in *Arabidopsis*, as we applied the scan to multiple species and wanted to avoid falsely labeling promoters as Coreless. Illustration of the regions scanned for each core promoter type is illustrated in Fig. 1e.

**Fig. 2.**
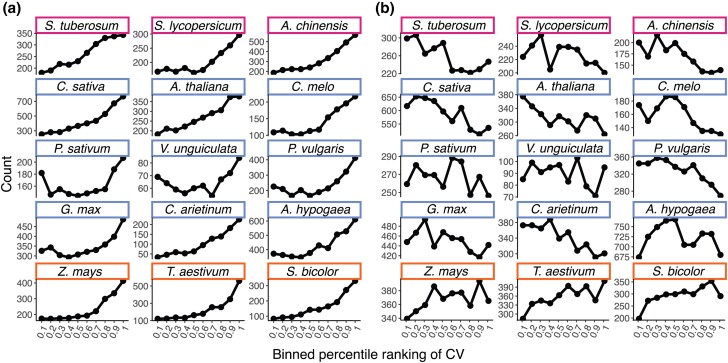
Distribution of relative specificity or uniformity of TATA-box-containing and Coreless promoters. Higher CV rankings indicate more specificity, while lower CV rankings indicate more uniformity. A random subsampling of 40% of promoters from each species are shown here. a) TATA-box containing promoters, and b) promoters termed Coreless as they lacked both TATA-box and Y-path motifs. Colors correspond to phylogeny shown in Fig. 1a.

The subset of promoters for each species was labeled as either TATA or Y patch. If a promoter did not contain either element, we labeled them as “Coreless.” It is important to note that the definition of Coreless promoters introduced by Yamamoto et al. (2009) is somewhat more strict than the definition used here, as they also screened for the relatively rare CA and GA core promoter elements. We then plotted the distribution of CV for each species, broken down by core promoter types (Fig. 2). Similar results for Y patch, Inr, and a random set of promoters that serve as a control are in Supplementary Fig. 1.

Using microarray data, Yamamoto et al. (2011) had found that Coreless promoters are underrepresented in genes that respond to stimulus (i.e. more constitutively expressed). However, we did not see the same trend until we removed the lowest expressing transcripts from the analysis (transcripts with an average of less than 1 read). These extremely low read counts are likely to be unreliable, and an analysis of the weak-expressing genes that we removed revealed that they bias toward higher CV when compared to the rest of the genes in the dataset (Supplementary Fig. 2). This same minimum read number requirement was then applied to the rest of the species.

Overall, the expected trend of TATA-box-containing promoters being overrepresented in conditionally expressed genes is observed across all the species analyzed (Fig. 2a). In contrast, the trend of Coreless promoters being associated with more uniformly expressed genes was weaker and only observed in a subset of the angiosperms. The monocots (*Z. mays*, *T. aestivum*, and *S. bicolor*) all exhibited a strong trend of Coreless promoters associating with conditionally expressed genes (e.g. those with higher CV values), along with an enrichment of Y patch-containing promoters being associated with uniform expression (Fig. 2b and Supplementary Fig. 1). This inverted pattern could be explained in 2 ways given that a promoter not labeled as containing a TATA-box or Y patch is labeled as Coreless. Under this classification scheme, an apparent enrichment by one category of promoters could reflect a surplus of that type of promoter in a particular CV ranking bin or a depletion of the other 2 promoter categories in that same bin. The latter explanation seems more likely for the Y patch promoters in monocots, but further experimental tests are required to fully resolve this question. The surprising pattern of Coreless genes “flipping” their behavior in monocots might also reflect an as-yet-undefined promoter element that is lumped into the Coreless category here. For example, there may be slight differences in TATA motif, as has been described for maize (Mejía-Guerra *et al.* 2015). Accounting for this known source of variation, we did not see any significant decrease in the Coreless trend toward conditionally expressed genes (Supplementary Fig. 1).

Our analysis was able to identify correlations between promoter type and expression pattern across many genes and led us to wonder whether the presence or absence of a specific core promoter type was sufficient to determine expression pattern. To test this hypothesis, we decided to focus on orthologous genes found across the species examined in this study (Fig. 1c). This approach allows us to test if changes in core promoter architecture during evolution led to changes in the uniformity of expression. We started by finding orthologs of *Arabidopsis* genes, as *Arabidopsis* has the most well-annotated genome and has 47,684 transcripts with a nonzero transcript count in at least one of the sampled tissues. Of this total, we retained only the primary transcripts of each nonmitochondrial and nonchloroplast genes, resulting in a final total of 26,842 genes. The top 5% most uniformly expressed and top 5% most conditionally expressed genes were selected based on CV, along with a randomly selected control set of equal size (*n* = 1,343 genes in each category). The sets of genes were used to query the Ensembl or Phytozome database for orthologs in the rest of the 14 species in our dataset (Goodstein *et al.* 2012; Cunningham *et al.* 2021). The orthologs were searched for in the database where their reference transcriptome was downloaded to ensure matching of the target transcript name with the transcript counts. Orthologs of *A. hypogaea, C. arietinum, and S. tuberosum* were found using Phytozome, and the remaining species were found in Ensembl.

**Fig. 3.**
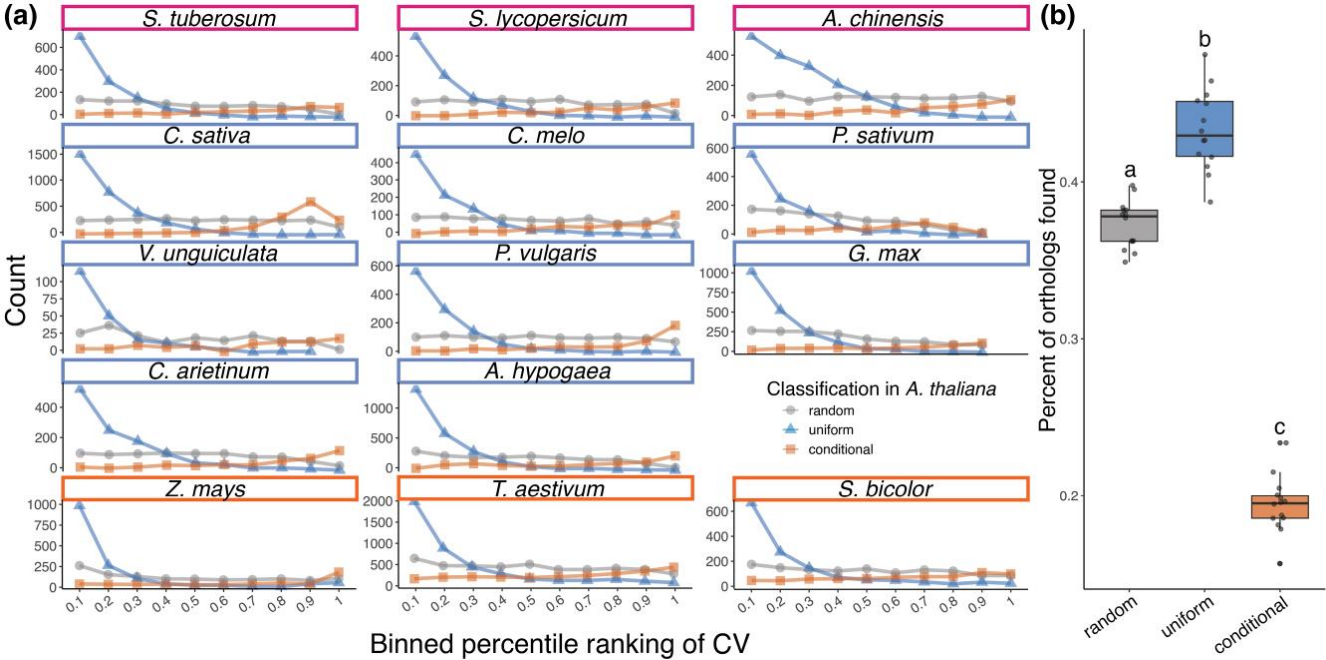
Genes that show uniform expression in *A. thaliana* tend to behave similarly in other species. a) Distribution of CVs for orthologs of uniformly expressed (triangle), conditionally expressed (square), or random (circle) *A. thaliana* genes. The color of boxes around species names corresponds to Fig. 1a. b) Percent of orthologs found for each set of *A. thaliana* genes for each species. Each dot corresponds to a single species. Statistical tests were performed by 1-way ANOVA followed by Tukey HSD. All 3 groups are significantly different from one another.

Orthologous genes tended to retain their expression pattern across species (Fig. 3a). While orthologs corresponding to the random set of *Arabidopsis* genes were spread quite uniformly across distribution of CV rankings, the orthologs of the top 5% uniformly expressed set of *Arabidopsis* genes were skewed heavily toward the more uniform, lower percentage CV rankings. The orthologs of the 5% most conditionally expressed set of *Arabidopsis* genes showed a more subtle skew toward higher CV ranking. This trend was more visible in some species than others, partially due to the overall lower gene counts. One notable trend was that the most conditionally expressed gene set retrieved significantly fewer orthologs compared to the random or most uniformly expressed gene sets (Fig. 3b). This is possibly because uniformly expressed genes are associated with more fundamental cellular functions and therefore more likely to be conserved across species (Klepikova *et al.* 2016a). Following a similar logic, conditionally expressed genes tend to be more tissue-specific and therefore are more easily lost during species divergence.

Even when looking at genes that fell at the tail ends of the expression uniformity distribution from *Arabidopsis*, we could find orthologs positioned across the full range of CV rankings (Fig. 3a). In other words, expression uniformity of a given gene can vary dramatically across species. These instances give us a unique opportunity to examine if the change in expression uniformity is predictive of changes in core promoter architecture. To investigate this further, we curated a set of evolutionarily related genes that showed this type of switching behavior. We limited our analysis to a set of 1:1 orthologs, where each species contributes a maximum of one gene for each orthologous gene set (Zahn-Zabal *et al.* 2020). This is to maximize confidence in evolutionary relatedness and minimize complications from gene duplications. Starting with the set of all the orthologs retrieved through Ensembl and Phytozome, we first filtered the target orthologs to count only the highest expressing transcript for each gene, thereby limiting each gene to a single representative transcript. We filtered the list of orthologs to include *Arabidopsis* transcripts that had only a single ortholog found in the transcriptome of each other species. We considered any target transcripts that crossed the 50th percentile in CV as “changing expression pattern,” and we limited the *Arabidopsis* transcripts to those where transcripts changed expression pattern in at least 2 different species. These changes were mapped onto the phylogenetic tree to identify clusters where changes could be associated with a specific phylogenetic node.

For the most promising *Arabidopsis transcripts,* de novo gene trees were built by performing BLAST searches against the rest of the species. When more than one ortholog was found in any of the species, that species was removed from the set (Fig. 1c). With our stringent selection criteria, 7 high-confidence orthologous gene groups were found with 3 *Arabidopsis* transcripts (AT3G17020.1, AT3G18215.1, and AT4G40045.1) that are from the top 5% uniformly expressed genes list and 4 *Arabidopsis* transcripts (AT1G04700.1, AT5G17400.1, AT5G18910.1, and AT5G20410.1) from the top 5% conditionally expressed genes list. A summary of the filters and numbers of target orthologs as well as *Arabidopsis* query transcripts left after each step can be found in Supplementary Table 2.

**Fig. 4.**
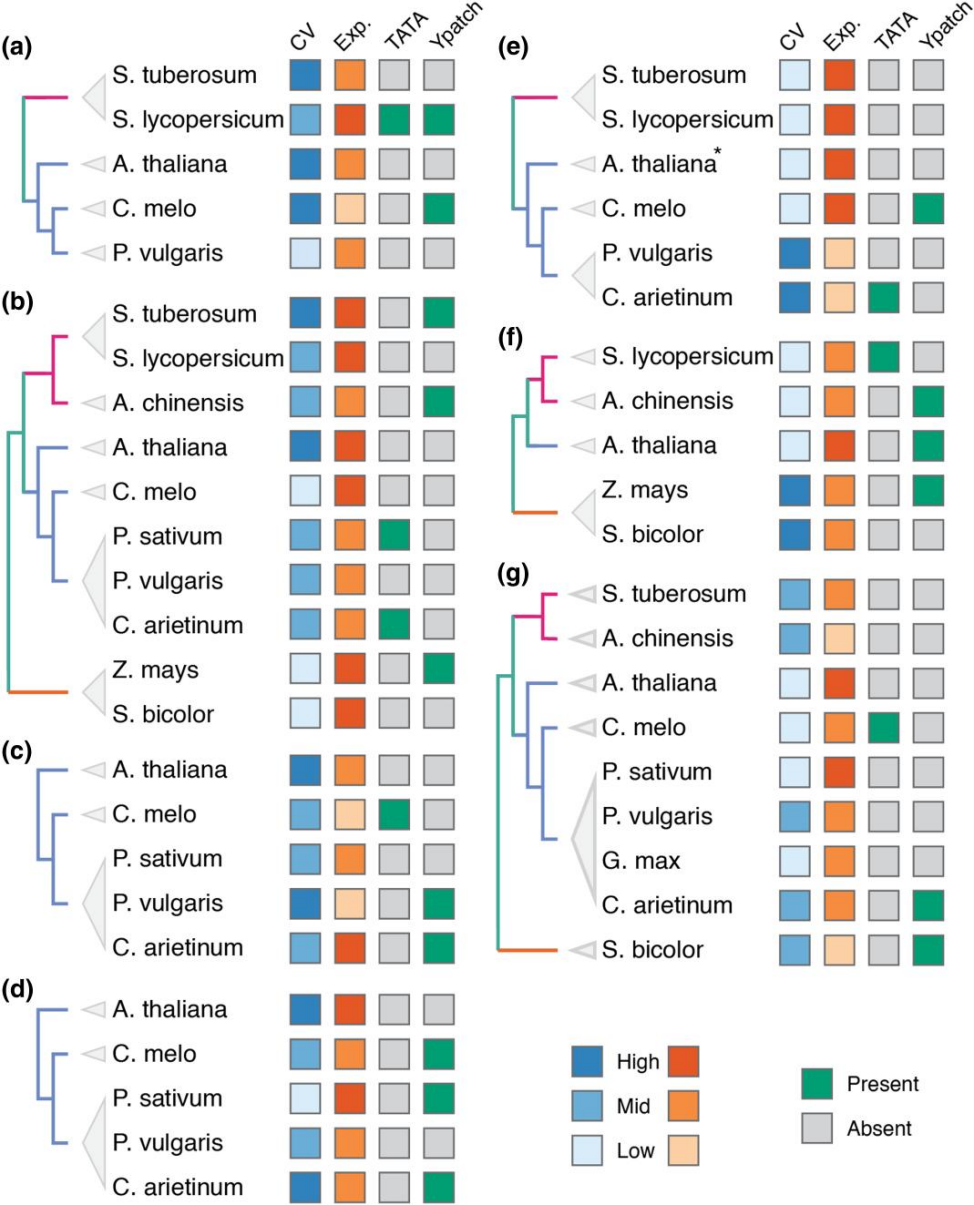
Individual gene trees where expression uniformity changes can be observed. a–d) The gene is conditionally expressed in *A. thaliana* but uniformly expressed in another species. e–g) The gene is uniformly expressed in *A. thaliana* but conditionally expressed in another species. The *Arabidopsis* genes in each group are as follows: a) AT1G04700, b) AT5G17400, c) AT5G18910, d) AT5G20410, e) AT3G17020, f) AT3G18215, GAT4G40045. CV and expression strength (Exp.) are grouped by percentile ranking of 0.66–1.00 (high), 0.33–0.66 (mid), or 0.00–0.33 (low) and color coded accordingly. Presence or absence (gray) of TATA and Y patch motifs is indicated. *A. thaliana has no identifiable core promoter as the intergenic region is only 8 bp.

The promoters for these 7 sets of orthologs were extracted, and TATA, Y patch, and Inr motifs were screened as described above (for clarity, this analysis will be referred to as motif scan) (Fig. 1d). In parallel, these promoters were also screened for TATA, Y patch, Inr, CA, and GA octamers as defined in Yamamoto *et al.* (2009) (octamer scan), and an illustration of the regions scanned for each octamers can be found in Supplementary Fig. 3. Comparing the 2 methods, the motif scan resulted in more identified core promoters due to its more relaxed parameters. Only 2 promoters were labeled as Y patch by the octamer scan but not the motif scan. A core promoter element was considered present if either method returned a positive result. The identification of the genes and a complete list of core promoter elements identified can be found in Supplementary Table 3. Within each orthologous gene group, changes in the presence of TATA or Y patch elements did not appear to correlate with changes in expression patterns (Fig. 4). In each group, there are examples of promoters having the same core promoter type but different expression patterns, as well as cases of promoters having the same expression pattern but different core promoter types. Since there were only 7 TATA-box-containing promoters (∼15.5% of the promoters), we were not able to observe instances where 2 related TATA-box-containing promoters have different expression patterns, but there are multiple instances where changes in presence of TATA motif did not change expression pattern. This result suggests that the presence or absence of a TATA or Y patch is not sufficient to change expression pattern.

## Discussion

Understanding the rules that govern the performance of natural promoters could inspire the construction of synthetic promoters that are able to retain their behavior over multiple generations in transgenic plants. Here, we mined RNA-seq atlases from 15 different angiosperms to extract patterns connected to the relative specificity or uniformity of gene expression across developmental stages and tissue types. We found that the previously observed trend that TATA-box-containing promoters are overrepresented in conditionally expressed genes is highly conserved. In contrast, the relative uniformity vs specificity of expression from Coreless promoters is not as well conserved. Coreless promoters from eudicots analyzed in this study were, in general, more highly associated with uniform expression patterns. Coreless promoters from monocot species, however, exhibited the opposite trend. In addition, we found that promoters tend to maintain their expression pattern across species, with the caveat that uniformly expressed genes are more likely to have identifiable orthologs when compared to conditionally expressed genes. Last, by tracking expression pattern and promoter type within the evolutionary trajectory of individual genes, we could test the hypothesis that promoter architecture is responsible for the level and pattern of gene expression. We found that none of the core promoter types screened for in this work is consistently associated with changes in expression pattern or strength. This suggests that while there may be a correlation between promoter architecture and transcription parameters, the underlying molecular mechanism that determines whether a gene is conditionally or specifically expressed remains unknown.

From a synthetic biology perspective, there are 2 major implications from the analysis described here. First, the hope of finding strong, constitutive natural promoters that work across diverse species may be even more challenging than we originally thought. For example, it is unlikely that there are natural promoter architectures that will work equally well as constitutive promoters in monocot and eudicot crops. Second, and more hopefully, our analysis suggests that the approach currently being taken by multiple labs for engineering synthetic promoters (Belcher *et al.* 2020; Brophy *et al.* 2022; Cai *et al.* 2020; Moreno-Giménez *et al.* 2022) is likely to find solutions that work well across species. The overall scheme of many of these groups is to take a core promoter region containing a TATA-box and then add natural cis-elements or synthetic transcription factor target sequences upstream of the core promoter to modulate expression strength or pattern. We found that the same core promoter could support widely varied expression patterns across evolution.

While the general trend that TATA-box-containing promoters are found in genes that are expressed in specific times and/or locations was highly conserved, close study of single gene phylogenies revealed that core promoters are not the determinant for expression pattern. The overall lack of pattern for TATA and Y patch motifs on the phylogenetic tree also suggests that the gain and loss of these promoter elements, at least in the genes studied here, are sporadic events that do not experience strong positive selection for maintenance. Our analysis leaves us with the question of why there is a discrepancy between the observed general preferences for core promoter types regarding expression uniformity but simultaneously a lack of contribution of core promoters to expression pattern, and whether there are mechanistic differences between Coreless and TATA promoters when they can achieve similar expression patterns. It is likely that constitutive expression can be achieved in at least 2 ways: by combining multiple tissue-specific elements that work together to achieve constitutive expression or by including “universal” elements that are broadly recognized across tissues. The analysis of the Cauliflower Mosaic Virus 35S promoter (p35S) showed that progressive deletion reduced promoter activity without affecting expression pattern and identified a short enhancer element that conferred constitutive expression (Odell *et al.* 1985; Fang *et al.* 1989; Hayashi *et al.* 1992). A more recent analysis of the p35S and other constitutive promoters, however, revealed multiple tissue-specific transcription factor binding sites (Cai *et al.* 2020). Performing a similar functional analysis on multiple Coreless promoters will be needed to determine whether the 2 classes of promoters achieve uniform expression using similar mechanisms. In addition, a more granular deletion analysis targeting individual cis-elements for both classes of promoters in multiple species, along with close examination of expression pattern, will be needed to fully map out promoter logic sufficiently to guide future engineering efforts.

## Data Availability

All scripts and datasets necessary to perform the analysis in the article, as well as supplemental data, are available at https://doi.org/10.5061/dryad.9w0vt4bmk.
